# Investigate the Role of Glutathione S Transferase (GST) Polymorphism in Development of Hypertension in UAE Population

**Published:** 2012-08-30

**Authors:** Kosar Hussain, Neveen Salah, Sahar Hussain, Sara Hussain

**Affiliations:** 1 Rashid Hospital, Dubai Health Authority, Dubai, United Arab Emirates; 2 Department of Biochemistry, Dubai Medical College, Dubai, United Arab Emirates; 3 UAE University, Faculty of Medicine and Health Sciences, Al Ain, United Arab Emirates; 4 Dubai Medical College, Dubai, United Arab Emirates

**Keywords:** Hypertension, Glutathione S-Transferase T1, Polymorphism, Genetic

## Abstract

**Background:**

GST is a family of enzymes that are important in protection of the body against oxidative stress.

**Objectives:**

Investigate the association between GSTT1 and GSTM1 polymorphism and hypertension.

**Materials and Methods:**

GSTT1 and GSTM1 genotypes were detected by PCR. The fragments were then analyzed by agarose gel electrophoresis.

**Results:**

There is no significant association between GSTT1 & GSTM1 polymorphism and hypertension (OR = 2.4, P > 0.05 and OR = 1.6, P > 0.05)

**Conclusions:**

GSTT1 & GSTM1 polymorphism can be considered a risk factor for hypertension.

## 1. Background

Essential hypertension is a complex multi-factorial disorder with many genetic, environmental and demographic factors contributing to this disorder. Several experimental and clinical studies have highlighted the role of oxidative stress in development of hypertension [1][2][3][4]. Our body possesses a number of protective antioxidant mechanisms to counteract the production of ROS. One such system is the family of Glutathione-S-Transferase (GST) that functions as detoxification enzymes [5]. GST activity has been demonstrated in vascular tissue, and represents a main cellular defense mechanism against oxidative injury [6]. The polymorphism in the GSTT1 and GSTM1 gene loci is caused by a gene deletion, causing GSTT1-0 (GSTT1 null) and GSTM1-0 (GSTT1 null) genotype respectively.

## 2. Objectives

The present study aimed to test the hypothesis that the loss of activity of the enzyme due to a deletion polymorphism in the GSTT1 and GSTM1 may affect the risk of developing hypertension. We have also studied other risk factors for hypertension.

## 3. Material and Methods

### 3.1. Subjects

The control group consisted of 33 non-hypertensive individuals with mean age 41.7 ± 13.6 years.The case group consisted of 30 hypertensive patients with mean age 40.1 ± 14 years. The study was conducted in the Biochemistry department in Dubai Medical College for girls. All subjects signed an informed consent to participate in the study. The hypertensive patients underwent a standardized evaluation consisting of a questionnaire, physical examination, and laboratory tests. Weight, height, waist and hip circumferences were measured. Body mass index (BMI) and Waist Hip Ratio (WHR) were calculated. Hypertension was defined as blood pressure ≥ 140/90 mmHg or current use of anti-hypertensive medication.

### 3.2. Anthropometric Measurements

Subjects with body mass index (BMI) ≥ 25 kg/m2 were considered positive for obesity as defined by World Health organization. Those with waist circumference (WC) > 88 cm for women and > 95 cm for men or with waist hip ratio (WHR) > 0.8 for women and > 0.95 for men were considered positive for abdominal obesity [7].

### 3.3. Laboratory Tests

Lipids and lipid fraction measurements were performed using routine enzymatic tests (DiaSys Kits) as previously described [8][9]

### 3.4. DNA Extraction

DNA was extracted from white blood cells by a salting-out method [10].

### 3.5. Analysis of GSTT1 and GSTM1 Genotype

For genotype analysis, the GSTT1 and GSTM1 were amplified by using multiplex polymerase chain reaction (PCR) protocol [11][12][13]. Polymerase chain reaction was done on 96 well Amp PCR System 9700 Thermocycler (Applied Biosystems). Primer sequences and PCR conditions were as follows (oligonucleotides were synthesized by Sigma Aldrich, Germany). For detection of GSTM1 polymorphism, the forward primer was 5’-GAACTCCCTGAAAAGCTAAAGC-3’ and reverse primer was 5’-GTTGGGCTCAAATATACGGTGG-3’. For detection of GSTT1 polymorphism the forward primer was 5’-TTCGTTACTGGTCCTCACATCTC-3’ and reverse primer was 5’-TCACGGGATCATGGCCAGCA-3’. In order to confirm that the PCR had worked in subjects homozygous for GSTM1 or GSTT1 gene deletion, one pair of primers was used as an internal control to amplify a 312 bp fragment of CYP1A1 gene: the forward primer was 5’GAACTGCCACTTCAGCTGTCT-3’, and the reverse primer was 5’CAGCTGCATTTGGAAGTGCTC-3’. Multiplex PCR mixture was carried out in a 50-μL reaction volume containing 100 ng of genomic DNA, 0.2 umol/L of each primer, 0.8 mmol/L dNTPs, 2.0 mmol/L MgCl2 in10% PCR buffer and 1.5U of DNA polymerase (Promega, UK). PCR involved an initial 2-min denaturation at 95°C, 30 cycles of denaturing at 94°C for 1 min, annealing at 64°C for 1 min and extension at 72°C for 1 min, with a final extension at 72°C for 5 min. Detection of different genotypes were done by 1.5% agarose gel electrophoresis, the presence of band 480 bp indicate the presence of GSTT1 while the presence of band of 215 bp indicate the presence of GSTM1.

### 3.6. Statistical Analysis

Statistical analysis was done with SPSS software version 11.0 (SPSS, Inc; Chicago IL). Difference in genotype prevalence and association between case and control group were assessed by the Chi-square test. Correlation coefficient, Odds Ratio (OR) and 95% CI were used to describe the strength of association.

## 4. Results

The distribution of genotypes of GSTM1 and GSTT1 in cases and control are shown in Table 1.

**Table 1:** Distribution of GSTM1 and GSTT1 Genotype Among Cases and Control

**Genotype**	**Cases (n = 30)**	**Control (n = 33)**	**Odds Ratio**	**95%CI**	**P-value**	**X^2^**
GSTM1 No. (%)						
Null	18(60)	16(48.5)	1.6	0.6-4.3	0.360	0.8
Non-null	12(40)	17(51.5)				
GSTT1 No. (%)						
Null	9(30)	5(15.2)	2.4	0.7-8.2	0.157	2.0
Non-null	21(70)	28(84.8)				
GSTT1 and GSTM1 No. (%)						
Null both ,	5(16.7)	2(6.1)	3.1	0.6-17.4	0.181	1.8
Others	25(83.3)	31(93.9)				

The results of anthropometric measurements & lipid profile among the cases and control are displayed in Table 2.

**Table 2:** Distribution of Anthropometric Measurements Among the Cases and Control

**Parameter**	**Control (n = 33)**	**Cases (n = 30)**	**OR**	**95% CI**	**P-value **	**X^2^**
BMI, No. (%)			2.5	0.9- 7.0	0.083	3
normal	17 (51.5)	9 (30)				
overweight and obese	16 (48.5)	21 (70)				
Waist-hip ratio, No. (%)			1.4	0.50- 3.7	0.560	0.3
normal	20 (60.6)	16 (53.3)				
abnormal	13 (34.9)	14 (46.7)				
TAG, No. (%)			1.4	0.5-3.7	0.532	0.4
< 150mg/dL	18 (54.5)	14 (46.7)				
≥ 150mg/dL	15 (45.5)	16 (53.3)				
TC, No. (%)			1.80	0.7- 4.9	0.250	1.3
< 200mg/dL	18 (54.5)	12 (40)				
≥ 200mg/dL	15 (45.5)	18 (60)				
HDL, No. (%)			1.4	0.5- 3.7	0.532	0.4
≥40mg/dL	18 (54.5)	14 (46.7)				
<40mg/dL	15 (45.5)	16 (53.3)				
LDL, No. (%)			1.70	0.5- 6.1	0.409	0.7
< 160mg/dL	28 (84.8)	23 (76.7)				
≥ 160mg/dL	5 (15.2)	7 (23.3)				

## 5. Discussion

Oxidative stress may contribute to the generation and/or maintenanceof hypertension via a number of possible mechanisms [14][15][16]. There is increasing interest in the role of GST polymorphism as a contributory factor to oxidative stress. Tang et al reported that subjects with GSTM1-0/GSTT1-0 had higher C-reactive protein and fibrinogen and lower total antioxidant status compared to patients with wild-type GSTM1/GSTT1 genes [17]. A recent study by Rybka et al. has shown that the glutathione-related antioxidant defense system was enhanced in elderly hypertensive patients who were receiving anti-hypertensive treatment [18]. On the other hand Marinho and colleagues demonstrated that although GST activity and total plasma glutathione levels were markedly decreased in hypertension but there is no correlation with the GSTM1 and GSTT1 deletion polymorphisms [19].In our study it was seen that null GSTM1 and GSTT1 were not a significant risk factor for hypertension. However in an Italian case-control study by Polimati et al. concluded that GSTT1 null individuals were significantly associated with increased risk of hypertension [P < 0.001; adjusted OR 2.24 (1.43-3.50)]. In the sub-analysis they have shown that the risk was significantly higher in female hypertensives [P < 0.001; adjusted OR 3.25 (1.78-5.95)] but not in male subjects [20]. Also Capoluongo et al. have published a similar study that showed GSTM1 null-genotype is a risk factor for hypertension among elderly subjects (OR = 2.25, 95% CI = 1.36–3.72;P = 0.005), while null GSTT1 genotype was a minor risk factor for hypertension (OR = 1.24, 95% CI = 0.67-2.29, P = 0.52). However, they failed to demonstrate that a combined GSTM1 and GSTT1 null-genotype act synergistically to increase the risk of hypertension [21]. Oniki et al. have also demonstrated that subjects who have combined GSTM1 and GSTT1 null genotypes have higher risk for hypertension (adjusted OR: 3.1; 95% CI: 1.0-9.5, respectively). [22]. Obese subjects are more prone to develop hypertension and various neurohormonal mechanisms are postulated to be involved [23][24]. Various clinical intervention trials have consistently found that weight loss effectively lowers blood pressure [25]. We recommend further studies to assess the role of other genes involved in anti-oxidant pathway.
